# Bilateral cervicoenamel projection and its management: A case report with lingual involvement

**DOI:** 10.4103/0972-124X.60233

**Published:** 2009

**Authors:** Nilofar B. Attar, Mangesh B. Phadnaik

**Affiliations:** *Department of Periodontics, Government Dental College and Hospital, Aurangabad - 431 001, Maharashtra, India*

**Keywords:** Cervical-enamel projection, furcation, odontoplasty, periodontal pocket, regeneration

## Abstract

Bacterial plaque has been implicated as the primary etiological factor in the initiation and progression of periodontal disease. Anatomic factors such as cervical enamel projections, enamel pearls, and developmental grooves are often associated with advanced, localized periodontal destruction. Enamel projections and pearls are mostly associated with molars. Enamel projections in the furcation areas of molars have no true attachment and are therefore highly susceptible to the creation of a deep periodontal pocket. A close association has been reported in the past between enamel projection and furcation involvement. Here, we present a case report with bilateral cervical-enamel projection and its management by flap operation with odontoplasty, and regenerative procedure by placement of graft in the osseous defect. Decreased pocket depth and a gain in clinical attachment level were observed on follow-up.

## INTRODUCTION

Periodontal disease is not a single entity nor is there a single causative factor that elicits a consistent periodontal response. The primary cause of gingival inflammation is bacterial plaque along with other predisposing factors. Dental plaque has been implicated as the primary etiological factor in periodontal disease. Host response to this etiological factor presents a wide range of responses. The irreversible damage follows the form of inflammatory periodontal disease when etiological factors remain undetected or ignored and their effects, over a period of time, surpass the resistance capacity of the host. It is of paramount importance to disclose and reverse the factors producing the changes early in the genesis of the disease process.

Additional local factors contributing to inflammatory and degenerative results include:

1. Factors propagating plaque retention and accumulation include calculus, topography of the root, inadequate restoration, overhangs, food impaction, orthodontics appliances, tooth position, iatrogenic factors, extraction of third molars, periodontal pockets, and dental caries.
2. Anatomic aberrances like palatoradicular grooves, cervical enamel projection, enamel pearls, cysts, foreign bodies, paltal rugae, etc.
3. Habits and self-inflicted injuries.
4. Mechanical factors include improper tooth brushing, use of abrasive dentrifices and other oral hygiene modalities like dental floss and oral lavage. Thermal and radiation factors include tissue burn due to hot food, electrosurgery, and biochemical factors include injury due to use of dental materials, tobacco, and chemical dessicants.

Developmental abnormalities such as cervical enamel projection, enamel pearl, or palatogingival grooves, may predispose the affected area to plaque accumulation, making oral hygiene procedure, scaling, and root planing difficult, and consequently, cause periodontal breakdown. “Cervical enamel projections (CEPs) are the focal apical extension of the coronal enamel beyond the normally smooth cervical margin on to the root of the tooth”. They are flat ectopic deposits of enamel that are triangular in shape and tapering in form, extending apically into the furcation area.

Cervical enamel projections on molars have been defined as a dipping of the enamel from the cemento-enamel junction toward the furcal area of molars.[1,2] Strong associations have been shown for the presence of cervical enamel projections and furcation involvement. These projections can affect plaque removal, can complicate scaling and root planing, and may be a local factor in the development of gingivitis and periodontitis. CEPs should be removed to facilitate maintenance.

The current article reviews the literature, stresses the importance of contributing factors in the initation and progression of periodontal disease, and presents a case report with bilateral cervical enamel projection and its successful management.

## REVIEW OF LITERATURE

Interest in root enamel goes back to the 1^st^ half of the 19^th^ century when detailed descriptions of enamel pearls and cervical enamel projections were presented. A treatise on the *Comparative anatomy of the teeth* (1840) with exquisite illustrations clearly indicates the presence of enamel extensions on both maxillary and mandibular molar in both humans and primates.[3]

Linderer's[4] article shows a clear representation of enamel that is seen extending into the bifurcation area of a mandibular molar. In 1949, Atkinson[5] first mentioned the possible relationship between cervical enamel projections (CEPs) and periodontal pocket formation. The most detailed clinical investigation of the enamel in the interradicular region was reported by Suzuki in 1958,[6] who described a particularly varied pattern of cervical enamel extension on molar teeth following removal of a thin layer of cementum from the root surface.

In 1967, a study by Leib *et al.*[7] showed no significant difference between the prevalence of furcation involvement on surfaces with CEPs compared with surfaces without CEPs. However, several other studies have shown a positive correlation between the prevalence of CEPs and a reduced level of periodontal attachment in the furcation area of molars.[2–6,7]

### Classification

Masters and Hoskins[1] suggested a classification system in 1964 that was based on the extent of cervical enamel projecting into the furcation area.

Grade I - The enamel projection extends from the cementoenamel junction of the tooth toward the furcation entrance.

Grade II - The enamel projection approaches the entrance to the furcation. It does not enter the furcation, and therefore, no horizontal component is present.

Grade III - The enamel projection extends horizontally into the furcation.

### Prevalence

1. The reported prevalence of CEPs has varied significantly among studies; overall mean values have ranged from 8.6 to 32.6% in molars.[1–3]
2. The prevalence of CEPs varied between the first, second, and third molars.
3. A higher prevalence of CEPs was found in the mandibular molars than in the maxillary molars.
4. The prevalence is highest for mandibular and maxillary second molars.
5. Cervical enamel projection has been reported on maxillary central incisors and premolars.[8]
6. Found frequently on buccal surface.
7. Grade II enamel projection has higher prevalence.

Zee and Bratthal[9] have studied the prevalence of cervical enamel projections and the correlation with furcation involvement in Eskimo dry skulls. The results showed a prevalence of 72% of CEPs among the 834 molars examined: 53% was grade III, 9% was grade II, and 11% was grade I. Mandibular molars had a higher prevalence of CEPs (78%) than did maxillary molars (67%).

### Significance

1. Atkinson[5] first mentioned in 1949 the possible relationship between CEPs and periodontal pocket formation.
2. Masters and Hoskins[1] later stated in 1964 that when CEPs extend into the root furcation, the fibers of the periodontal ligament do not have true attachment to the tooth in the area of the enamel extension. They considered CEPs to be a potential etiological factor in periodontal disease.

### Treatment

The enamel projection may be eliminated down to the crestal bone level by ‘saucerization.[9] Osteoplasty, odontoplasty, or regenerative procedures may be required to treat the osseous defect due to cervico-enamel projections.

## CASE REPORT

**Figure 1:**
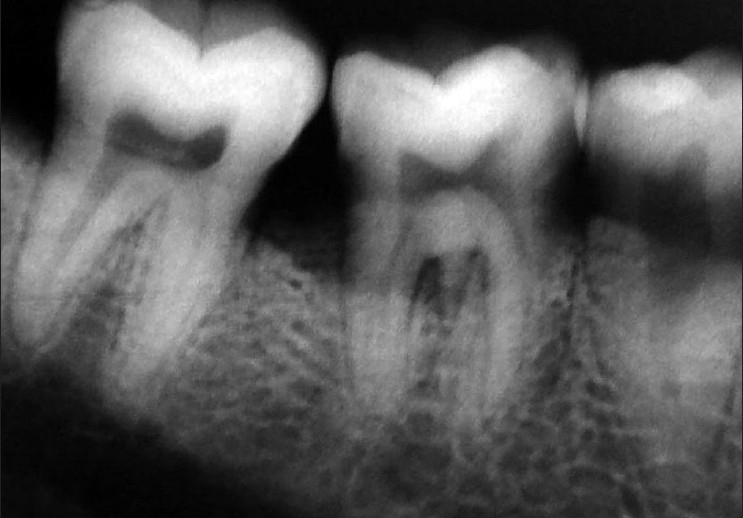
Preop radiograph (right side)

**Figure 2:**
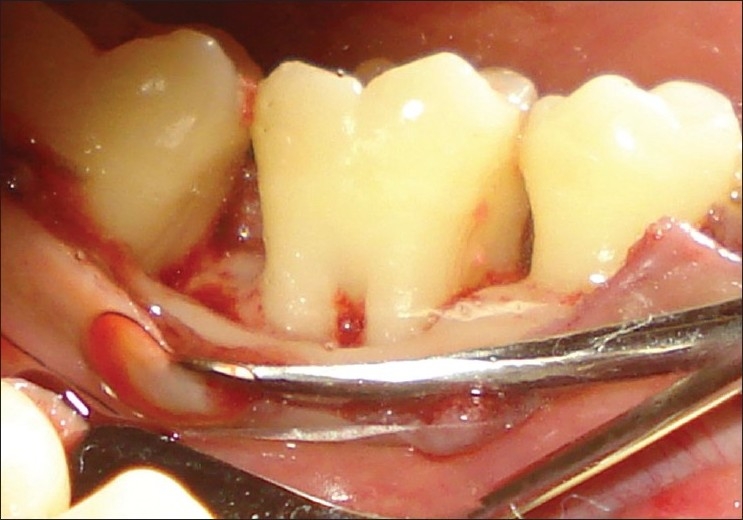
Cervicoenamel projection on right side (mirror view)

**Figure 3:**
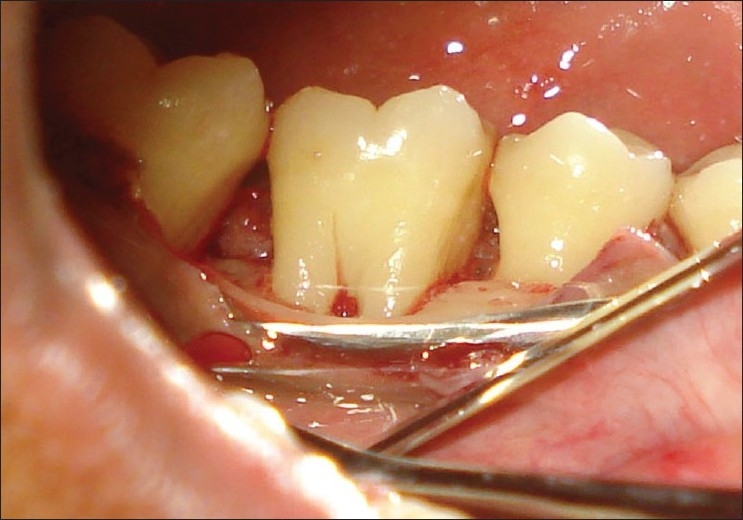
After odontoplasty (right side mirror view)

**Figure 4:**
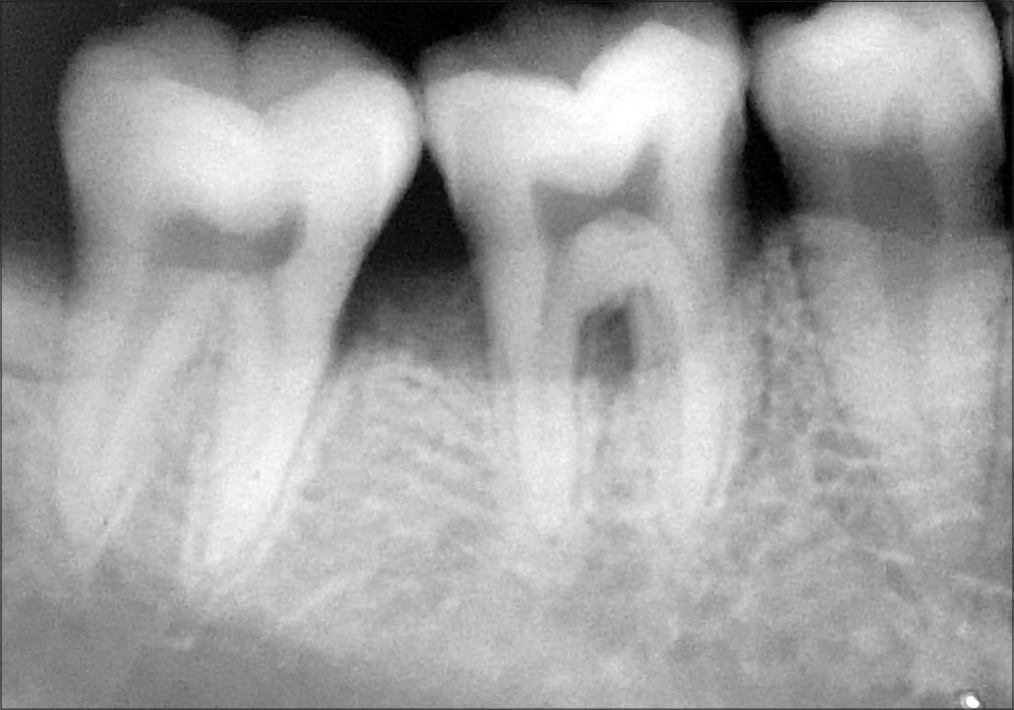
Postop radiograph (right side)

**Figure 5:**
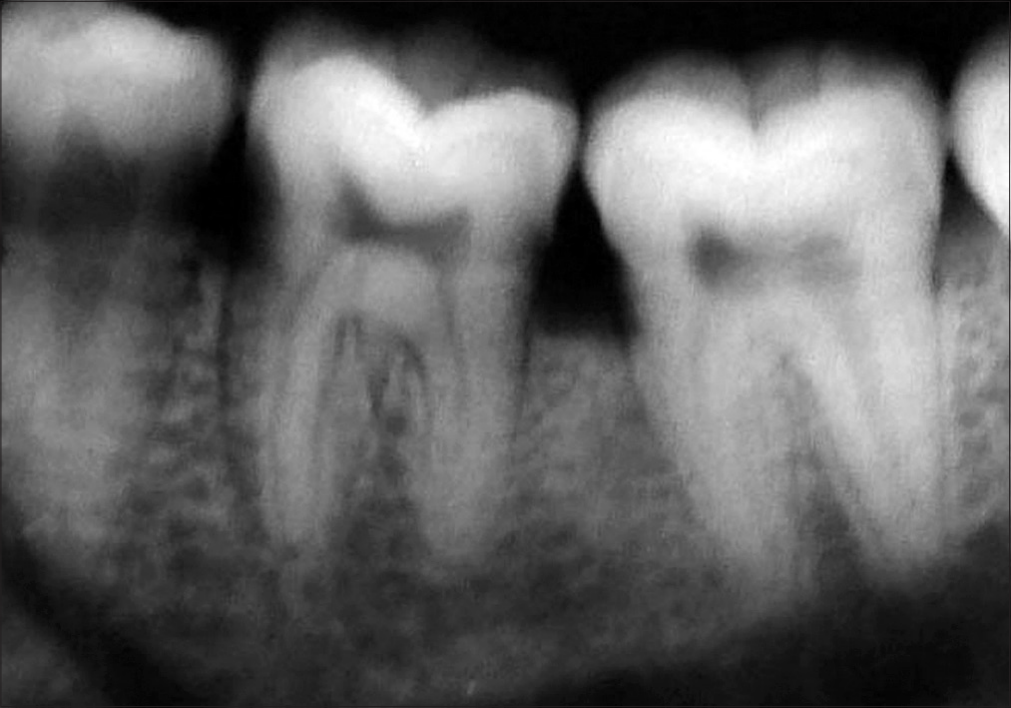
Preop radiograph (left side)

**Figure 6:**
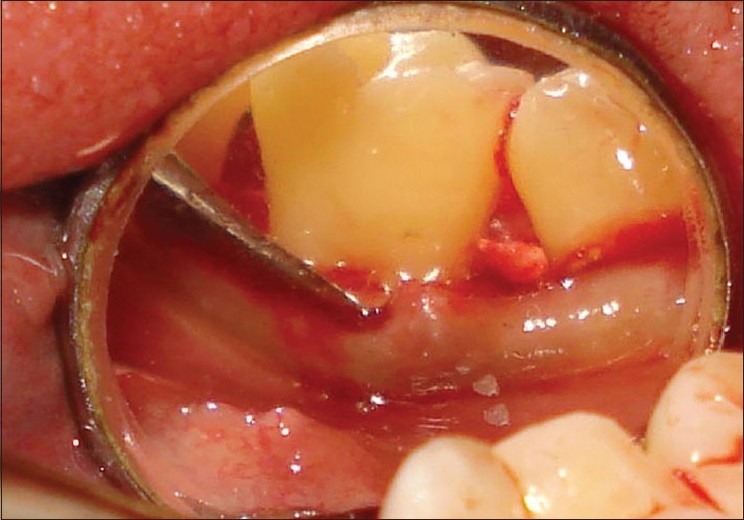
Cervicoenamel projection on left side (mirror view)

**Figure 7:**
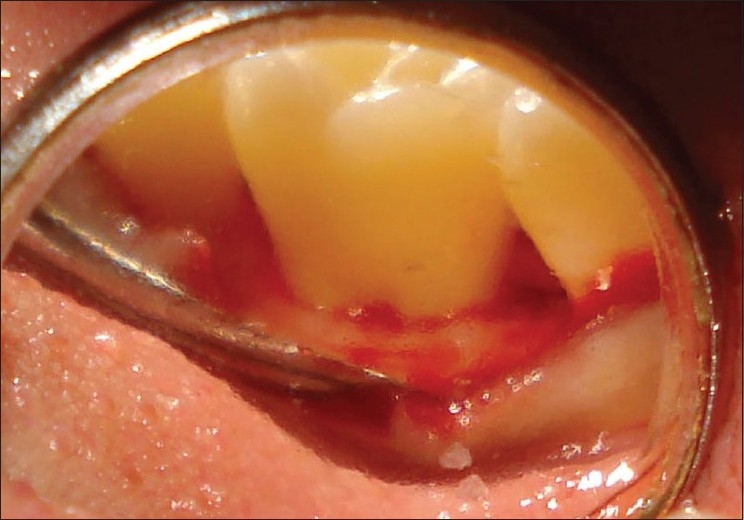
After odontoplasty (left side mirror view)

**Figure 8:**
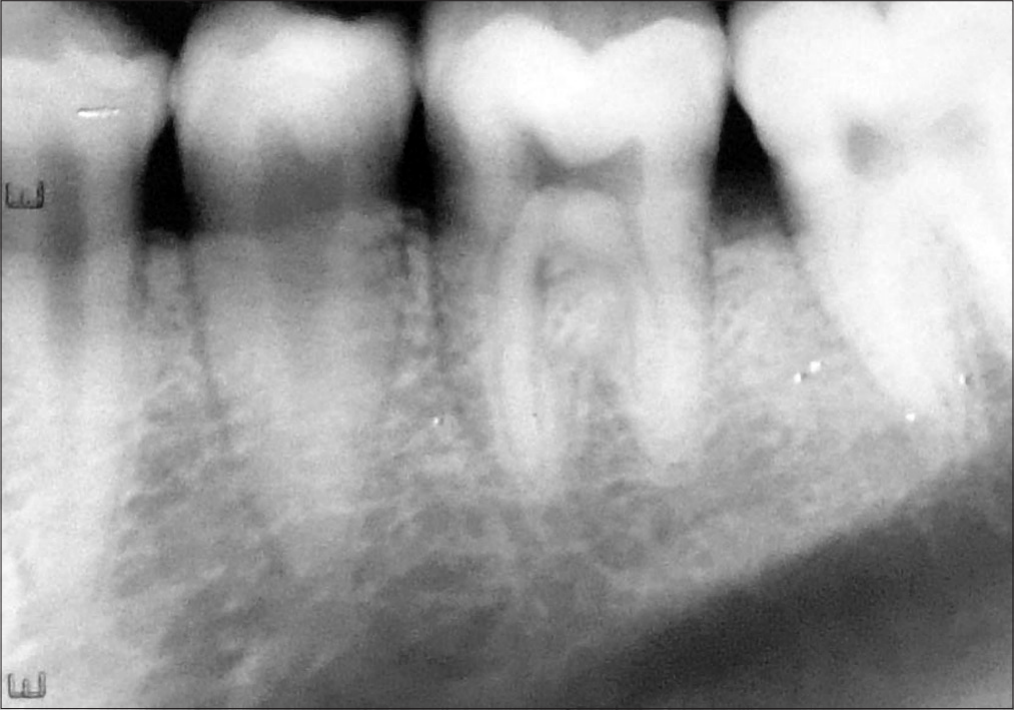
Postop radiograph (left side)

A 24 year-old boy reported to the Department of Periodontics at the Government Dental College and Hospital, Aurangabad, with the chief complaint of bleeding gums. A diagnosis of localized chronic periodontitis was made on the basis of history and clinical and radiological examination results. Clinical examination showed deep periodontal pockets on the lingual surface with Grade II furcation involvement in the right and left mandibular first molars. Radiographic examination showed cervical enamel projection; phase I therapy was completed. Open flap debridement revealed Grade II cervical enamel projection on the lingual surfaces of #36 and #46. The cervical enamel projection was eliminated down to the crestal bone level by odontoplasty followed by a regenerative procedure. The sixth month's follow-up of the patient showed clinical attachment gain and decreased periodontal probing depth [Figures 1–8].

## DISCUSSION

Cervical enamel projection is the most common anatomical variation in molar teeth. Cervical enamel extensions occur at the mid-cervical line and consist of a V-shaped, smooth or rough extension of the enamel over the root. Cervical enamel extensions are associated with chronic periodontal infection in furcal areas and with inflammatory cysts known as buccal bifurcation cysts. The buccal bifurcation cyst is most commonly seen in younger children. CEPs can be detected on routine examination with an explorer or probe, radiograph or during surgical intervention.

A higher prevalence of CEPs was found in mandibular molars than in maxillary molars.[10] The prevalence of CEPs varies between the first, second, and third molars and is the highest for the mandibular and maxillary second molars. The lowest prevalence was seen in maxillary third molars. Some authors have reported a greater prevalence of furcation involvement in mandibular first molars.[4] The reported prevalence of CEPs has varied significantly among studies: Overall mean values have ranged from 8.6 to 32.6%.[1–8] Hou and Tsai[10] and Swan *et al.*[2] reported Grade I CEPs to be the most common whereas Machtei and Waentein[11] reported Grade II CEP to be the most prevalent (34.8%). Wang and coworkers[12] have reported that 62.5% of teeth with mandibular bifurcation defects exhibit CEPs. They concluded that mandibular molars with class II bifurcation defects are very likely to have CEPs.[7] Therefore; it would appear that the presence of a CEP is a cofactor in the etiology of isolated furcal invasions.

CEPs are probably related to more rapid progression of pocket formation because of their anatomy and location. The enamel covering of the CEP precludes an organic connective tissue attachment due to which a hemidesmosomal attachment probably exists in the region of the CEP, which may be less resistant to breakdown by bacterial plaque. Once breakdown occurs, rapid progression of the disease becomes more likely because the projection morphology of the cervical enamel allows the retention of microbial dental plaque. In addition, the region's inaccessibility to cleansing and its proximity to the furcation could predispose the pocket to further furcation invasion.

A few reports have provided some guidelines for the treatment of multirooted teeth with furcation involvements associated with prominent enamel projections. One of the techniques is the incorporation of projection flattening or removal, into surgical procedures such as the modified Widman flap or the exicisional new attachment procedure combined with furcationplasty. This would contribute to the promotion of collagen fiber attachment in grade I and shallow grade II furcal lesions. In addition, opening the furca for access to plaque control with special brushes or cleaners is another adjunctive approach for the management of grade III and grade IV furcal lesions.

In the present case, the most viable therapeutic approach was thought to be an exploratory surgical procedure to determine the nature of the entity and to render appropriate treatment. Intraoral periapical radiographs showed cervical enamel projections extending below the crest of the bone. Open flap debridement revealed grade II cervical enamel projection with the lingual surface of the right and left mandibular first molars. The actual treatment performed was uncomplicated and consisted primarily of the removal of the anomaly along with a regenerative procedure. This resulted in a smooth surface of the hard tooth structure, which allowed proper adaptation of the soft tissue flap. A reduction in inflammation and probe penetration occurred after periodontal therapy.

## CONCLUSION

Cervical enamel projections might be considered a secondary etiological factor in periodontal breakdown and attachment loss. Although at greater risk for breakdown, mandibular teeth with CEPs should be considered good candidates for regenerative procedures that target bifurcation defects.
